# The Impact of Negative Social Feedback on Wanting and Liking of Food Pictures in Anorexia Nervosa

**DOI:** 10.1002/erv.70012

**Published:** 2025-07-16

**Authors:** Ludovica Natali, Valentina Cardi, Chiara Tosi, Enrico Collantoni, Chiara Caulo, Francesca Fontana, Alessandra Sala, Enrico Ceccato, Palmiero Monteleone, Angela Favaro, Valentina Meregalli

**Affiliations:** ^1^ Department of General Psychology University of Padua Padova Italy; ^2^ Department of Neurosciences University of Padua Padova Italy; ^3^ Padua Neuroscience Center University of Padua Padova Italy; ^4^ Department of Medicine Surgery and Dentistry ‘Scuola Medica Salernitana’ Baronissi Università di Salerno Salerno Italy; ^5^ Centro Provinciale di Treviso per i Disturbi del Comportamento Alimentare Treviso Italy; ^6^ Eating Disorders Unit Ospedale San Bortolo Vicenza Italy

**Keywords:** computerised, criticism, eating, restriction, social rejection

## Abstract

**Objective:**

Negative emotional states have been found to predict food cravings and consumption in the general population. People with a persistent tendency to restrict food intake, however, might be eating less when sad, angry, or stressed. In this study, the impact of inducing a negative emotional state through social exclusion on wanting and liking of food pictures was explored in patients with anorexia nervosa.

**Method:**

43 patients with anorexia nervosa and 22 healthy controls completed a computerised social rejection and food appraisal task. Participants viewed short videos in which a stranger made either a negative comment directed toward them or a neutral comment. After each video, participants rated their affective state, as well as their wanting and liking for pictures of high‐calorie foods.

**Results:**

Participants in both groups experienced greater negative affect after viewing the negative videos compared to the neutral ones. They also reported lower wanting for high‐calorie foods following negative videos, while no significant effects were observed for liking scores. Interestingly, patients with higher body mass index exhibited a greater reduction in both food wanting and liking following negative videos.

**Conclusions:**

These results suggest that negative social interactions can exacerbate restrictive behaviours, especially in patients who are recovering weight during treatment.

## Summary

- Negative social feedback reduced food wanting in both anorexia nervosa patients and healthy controls.
- Body mass index moderated the effect, with patients with higher BMI presenting greater reductions in food wanting and liking.
- Social rejection may reinforce restrictive eating, particularly in patients recovering weight during treatment.

## 1 Introduction

Anorexia Nervosa (AN) is a psychiatric disorder marked by extreme and sustained food restriction (Heaner and Walsh 2013). Although restriction is believed to be mainly driven by patients' desire and intention to lose weight, eating behaviours are influenced by a variety of factors, beyond individuals' explicit goals (Emilien and Hollis 2017). One of such factors is the body's effort to maintain a stable internal environment, which is determined by biological and genetic variables and defined as ‘homoeostasis’ (Gale et al. 2004; Saper et al. 2002). Also, psychological, socio‐economic, and cultural aspects play a substantial role in regulating food intake (Emilien and Hollis 2017; Zorbas et al. 2018). Individuals' need to belong to a social group, for example, can motivate to adapt one's own food intake to what peers eat, to both affiliate and reduce uncertainty (Cruwys et al. 2015). Moreover, social interactions are among the most influential determinants of both positive and negative emotions, which in turn can trigger specific eating patterns. It has been repeatedly observed that following a negative social experience, such as being excluded or criticised by others, healthy participants usually report a larger consumption of highly palatable foods compared to participants in a control (social inclusion) condition (Baumeister et al. 2005; Hayman et al. 2015). This evidence is consistent with research showing that negative emotions or stress can trigger an increase in food consumption, with a preference for consuming highly caloric and processed foods (e.g. snacks, fast food; a process also known as ‘emotional eating’) (Cardi et al. 2015; OLIVER and WARDLE 1999). A possible explanation is that negative emotions and stress activate the reward system and increase reward pursuit as an attempt to reduce negative feelings (Adam and Epel 2007).

At least two different components of the reward response have been characterised in the scientific literature, ‘liking’ and ‘wanting’ (Nguyen et al. 2021), which interact and yet rely on different and dissociable neural circuits (Morales and Berridge 2020). ‘Liking’ refers to the hedonic impact of pleasant stimuli, whereas ‘wanting’ refers to the motivation to pursuit a reward (Nguyen et al. 2021). Interestingly, the latter has been implicated more in the consumption of highly palatable foods in response to negative emotions (Lemmens et al. 2011; Pool et al. 2015). Pool et al. (2015) observed that after stress induction, participants made more efforts to achieve a chocolate reward, compared to participants in the non‐stress condition. Also, the increase in ‘wanting’ was not associated with higher levels of pleasure once the reward was experienced. Stress and negative emotions might thus promote the consumption of highly caloric and palatable foods by increasing the craving, ‘wanting’, for these stimuli. This seems particularly prominent in individuals with overweight or obesity, restrained eaters, and those suffering from binge eating symptoms (Cardi et al. 2015; Salvy et al. 2011).

On the opposite, in individuals affected by anorexia nervosa, it has been proposed that stimuli that usually activate the reward system, such as high calorie and palatable foods, seem to lose their natural incentive value and illness compatible stimuli, such as low‐calorie foods, physical activity, or restriction become rewarding instead (Keating et al. 2012; O’Hara et al. 2015). Accordingly, it has been observed that both explicit and implicit ‘wanting’ and ‘liking’ for high‐calorie foods are significantly reduced in patients as compared to controls (Cowdrey et al. 2013). It is therefore possible that in anorexia nervosa, acute stress and negative emotions elicit a further decrease in the ‘wanting’ for palatable foods and an increase in restricting behaviours, which is opposite to what is observed in healthy controls. Self‐report studies conducted so far are consistent with this hypothesis, as patients with anorexia nervosa report eating less than usual when being sad, angry, anxious or stressed (Meule et al. 2019; Reichenberger et al. 2021). In an ecological momentary assessment study, results showed that higher daily ratings of negative affect are associated with a greater likelihood of dietary restriction on subsequent days (Engel et al. 2013). However, no study to date directly induced a negative emotional state in patients with anorexia nervosa to evaluate its effect on ‘wanting’ and ‘liking’ for palatable foods.

Social situations can be a powerful source of negative emotions for patients, who display a heightened interpersonal sensitivity, and therefore a greater vulnerability to negative social interactions and rejection compared to healthy peers (Cardi et al. 2013; Rowlands et al. 2021). It could thus be hypothesised that perceived social exclusion and criticism might induce negative emotions in patients with anorexia nervosa, and therefore negatively influence food appraisals (i.e., reduce food liking and wanting). In the present study, participants with anorexia nervosa were asked to observe short videos displaying a stranger making a negative comment toward the person, or a neutral comment. The negative comment either related to the person's physical appearance or their personality. Following the presentation of each video, participants were exposed to a series of pictures of highly caloric foods and asked to rate the ‘liking’ and ‘wanting’ for these stimuli. The content of the videos was the same as that used in previous works assessing emotional responses to criticism in patients with eating disorders (Chami et al., personal communication (Rowlands et al. 2022). However, their impact on food appraisal has not been investigated until now. Consistently with previous studies, it was expected to observe an overall increase in food‐related ‘wanting’ in the healthy control group and an overall reduction in ‘wanting’ of high‐calorie foods in participants with anorexia nervosa, following induction of a negative affective state compared to the neutral condition. However, considering the heterogeneity in the presentation of eating disorder symptoms, and the relevance of illness severity on cognitive and affective processes (Tenconi et al. 2021), exploratory analyses were conducted based on the potentially significant associations between illness severity indexes (body mass index—BMI ‐ and eating disorder psychopathology, social anxiety) and task performance (i.e., food appraisal).

## 2 Method

### 2.1 Participants

Patients were recruited from daycare services at the Eating Disorder Units of Padova, Vicenza, and Salerno, in Italy (Northeast and South Italy). They met the DSM‐5 criteria of anorexia nervosa (American Psychiatric Association 2013), and their diagnosis was confirmed by an expert clinician. Healthy participants were recruited from the general population, through social media adverts and personal contacts. Inclusion criteria for all were: (1) female gender, (2) 14 years or older, (3) fluency in Italian. Exclusion criteria were: (1) self‐reported diagnosis of neurological disorders, (2) self‐reported diagnosis of psychosis or substance abuse disorders, (3) visual/hearing impairments not corrected by glasses/ear implants. Additional inclusion criteria for healthy controls were: (1) a body mass index (BMI, kg/m2) between 18.5 and 24.9, (2) a score lower than 2.8 on the global scale of the Eating Disorder Examination Questionnaire (EDE‐Q) (Mond et al. 2008), (3) a score lower than 75 on the global score of the Liebowitz Social Anxiety Scale (LSAS) (Baroni et al. 2023). Additional exclusion criteria for the same group were (1) self‐reporting a current or past diagnosis of mental disorder, (2) receiving a pharmacological treatment for a mental disorder condition.

The sample size was determined based on an a priori power analysis performed on G*Power (Faul et al. 2007). The analysis indicated that a total of 38 participants would be needed to detect a significant group by video content interaction with a small effect size (Cohen's *f* = 0.15, corresponding to *d* = 0.2 and *η* ^2^_p_ = 0.01) with a statistical power of 1−β = 0.80, at a significant level *α* = 0.05 and assuming a correlation among repeated measures of *r* = 0.8.

Written informed consent was provided by all participants. Consent was provided by parents or legal guardians for underage participants. The study was approved by the ethical committee of the hospital of Vicenza, in Italy (reference number: 1831) and was conducted in accordance with the latest version of the Declaration of Helsinki.

### 2.2 Procedure

Patients were tested at the hospital where they were receiving daycare, in Italy. Daycare for eating disorders, across the centres involved in the study, includes the following components: individual psychological therapy (based on principles of cognitive‐behaviour therapy), nutritional counselling, assisted meals, group activities (e.g., psychotherapy, art therapy, music therapy, relaxation protocols) and psychoeducation for family members. After signing the informed consent, participants completed an online survey via Qualtrics (https://www.qualtrics.com) which included answers to demographic questions and the following self‐report questionnaires: (1) the Eating Disorder Examination Questionnaire (EDE‐Q, Calugi et al. 2017; Fairburn and Beglin 1994), and (2) the Liebowitz Social Anxiety Scale (LSAS; Baroni et al. 2023). Also, they completed a computerised task on Opensesame (Mathôt et al. 2012), the ‘Social rejection and food appraisal task’ (see Section 2.3.2). Healthy controls completed the same questionnaires and computerised task online, using the platform www.gorilla.sc. Patients' weight and height were retrieved from clinical records at the hospital and used to compute the BMI. Since the control participants were recruited online, their BMI was computed based on self‐reported weight and height.

### 2.3 Materials

#### 2.3.1 Self‐Report Questionnaires

*Eating Disorder Examination Questionnaire (EDE‐Q)* (Calugi et al. 2017; Fairburn and Beglin 1994). The EDE‐Q is a 28‐item questionnaire assessing eating disorder psychopathology. Each item is scored on a scale from 0 to 6 and higher scores indicate greater severity. It comprises four subscales (restraint, eating concern, shape concern, weight concern) and a global score. The Cronbach's alpha of the subscales and the global score in this study was 0.9.

*Liebowitz Social Anxiety Scale (LSAS)* (Baroni et al. 2023; Heimberg et al. 1999). The LSAS is a 24‐item scale for the assessment of social anxiety. Each item is scored twice on a scale from 0 to 3 for the assessment of ‘fear’ and ‘avoidance’. Higher scores indicate greater severity. The Cronbach's alpha of the subscales and the total score in this study was 0.9.

#### 2.3.2 Social Rejection and Food Appraisal Task

In this task, participants are presented with 16 short videos lasting approximately 2 s and developed by amatorial actors. Each video features a young adult speaking a sentence directed to the participant. In half of the videos, the content of the sentence is meant to be emotionally neutral (e.g., ‘most high schools have a music band’), while in the other half, the content is a negative comment based on appearance (e.g., ‘you always look so unkempt’) or more general personal characteristics (e.g., ‘you are a boring person’). The neutral and negative videos are presented in two separate blocks. Block order is counter‐balanced between participants, and a two‐minutes break is inserted between blocks.

**FIGURE 1:**
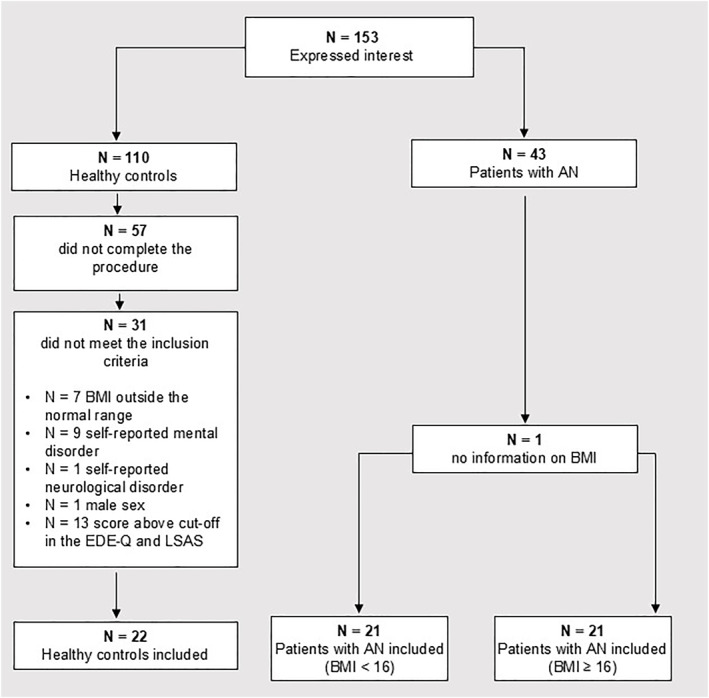
A consort diagram describing the flow of participation to the study. AN is Anorexia Nervosa; BMI is Body Mass Index (kg/m^2^); EDE‐Q is the Eating Disorder Examination Questionnaire; LSAS is the Lebowitz Social Anxiety Scale.

Before the presentation of each video, a fixation point is displayed in the middle of the screen for 2000 ms. At the end of each video, participants are asked to rate their current emotional state using the nine‐point Self‐Assessment Manikin (SAM) scale for valence (Bradley and Lang 1994). The SAM figures range from a frowning‐unhappy Figure 1, very unpleasant) to a smiling‐happy figure (9, very pleasant). Subsequently, they are presented with a sequence of five pictures depicting highly caloric and palatable foods, lasting 2000 ms each, and rate the ‘wanting’ and ‘liking’ toward the food pictures displayed on a scale from 1 to 5, answering the following questions: (1) ‘How much would you want to eat the foods you have just seen?’, (2) ‘Imagine eating the foods you just saw, how much would you like them?’. All food pictures were selected from the ‘food.pics’ database (Blechert et al. 2019).^1^ We selected images of foods commonly found in Italian cuisine (e.g. pizza, ice‐cream). All the selected items had a palatability rating of more than 50 in the Food‐Pics_Extended image database while considering mean scores for female omnivores (Blechert et al. 2019; Labontè et al., 2024) and they had a relatively high energy density (mean = 271.46 kcal/100 g). The phrasing of the liking and wanting questions was developed in consultation with a group of patients and therapists, and the wording that seemed most effective in eliciting stronger feelings of wanting and liking towards the pictures was selected.

### 2.4 Statistical Analyses

Data were analysed using *R* studio (R Core Team 2023). The outcome variables were affective valence, wanting and liking as rated during the Social rejection and food appraisal task. The independent variables were group (patients vs. healthy controls) and video content (negative vs. neutral). The analyses included three steps:

1. Linear Mixed Effects Models (LMMs) were computed to assess the differential impact of negative social evaluation on valence and food wanting and liking in patients with anorexia nervosa and healthy controls. The independent variables were included as fixed effects and their interaction was considered. The participants' ID was included as random factor. Age was also included in the models, as the groups were not age matched.
2. Spearman's rho correlation analyses were conducted to examine whether BMI, eating disorder psychopathology (EDE‐Q) and social anxiety symptoms (LSAS) were related to differences in valence, wanting and liking between conditions (negative—neutral) in the patient group. These analyses revealed a significant association between BMI and changes in wanting and liking.
3. LMMs were re‐conducted for the patient group to investigate how the video content differentially affected wanting and liking of high‐calorie foods based on BMI. Patients were divided in two groups: patients with BMI ≥ 16, patients with BMI < 16. The cut‐off value of 16 for BMI was selected based on the DSM‐5 criteria (American Psychiatric Association 2013), which distinguish between moderately underweight (BMI ≥ 16) and severely underweight (BMI < 16).

LMMs were fitted using the ‘lmer’ function, lme4 package (Bates et al. 2015) and explored using the ‘Anova’ function type three, car package (Fox et al. 2019).All continuous variables were centred and scaled. Variance Inflation Factors (VIF) were calculated with the ‘vif’ function, car package (Fox et al. 2019) to measure collinearity between predictors. Contrasts were performed with the ‘emmeans’ function, emmeans package (R Core Team 2017). All factors showed low collinearity, with values below 3 (Hair et al. 2010).

## 3 Results

### 3.1 Demographic and Clinical Characteristics

One hundred fifty‐three participants (110 healthy controls and 43 patients) expressed interest in participating in the study and were assessed for eligibility. Eighty‐eight controls were excluded: 57 did not complete the task; 31 did not meet the inclusion criteria (seven reported a BMI outside the normal range, nine reported suffering from a mental disorder, one reported suffering from a neurological disorder, one was a male, and 13 scored above cut‐off on the EDE‐Q or LSAS) (Baroni et al. 2023; Mond et al. 2008). The final sample included 65 participants: 22 controls and 43 patients. The flow of participation in the study is described in Figure 1.

Participants' sociodemographic and clinical variables are reported in Table 1. Groups differed significantly on age, years of education, BMI, eating disorder psychopathology and social anxiety symptoms (all *p* < 0.001, large effect sizes). Healthy controls were older, had a higher level of education and exhibited lower levels of eating disorder psychopathology and social anxiety symptoms. Patients reported a BMI in the moderately underweight range and clinically significant levels of eating disorder psychopathology (Calugi et al. 2017) and social anxiety (Baroni et al. 2023).

**TABLE 1:** Participants' demographic and clinical variables.

Variables	*M* (SD) or frequency (%)		*U*	*p*	*r*_*rb*_
	Controls (*n* = 22)	Patients (*n* = 43)			
Age^a^	21.59 (3.07)	18.70 (5.18)	691.500	0.001	0.497
Education (years)	15.00 (3.07)	11.58 (3.26)	741.500	< 0.001	0.568
Occupation (student vs. other)	20 (90.91%)	40 (93.02%)	—	—	—
Body mass index (kg/m^2^)	20.89 (1.19)	16.14 (1.45)	911.000	< 0.001	0.972
EDE‐Q restraint^a^	0.62 (0.78)	3.54 (1.71)	77.500	< 0.001	−0.836
EDE‐Q global score^a^	0.77 (0.65)	4.02 (1.27)	30.000	< 0.001	−0.937
LSAS fear scale^a^	18.50 (5.74)	45.26 (15.64)	64.500	< 0.001	−0.864
LSAS avoidance scale^a^	12.59 (5.55)	36.14 (16.34)	122.000	< 0.001	−0.742
LSAS total score^a^	31.09 (9.72)	81.40 (30.87)	76.000	< 0.001	−0.839

*Note: N* = 65. Participants' demographic and clinical variables expressed as means (standard deviations) or frequencies (%), *p‐values* from Mann‐Whitney test, effect sizes are given by the rank biserial correlation.

Abbreviations: DERS, difficulties in emotion regulation scale; EDE‐Q, eating disorder examination questionnaire; LSAS, liebowitz social anxiety scale.

^a^ *N* = 64.

### 3.2 The Impact of Negative Social Evaluation on Affective Valence, Wanting and Liking of High Calorie Foods in Patients With Anorexia Nervosa and Heathy Controls

#### 3.2.1 Valence

**FIGURE 2:**
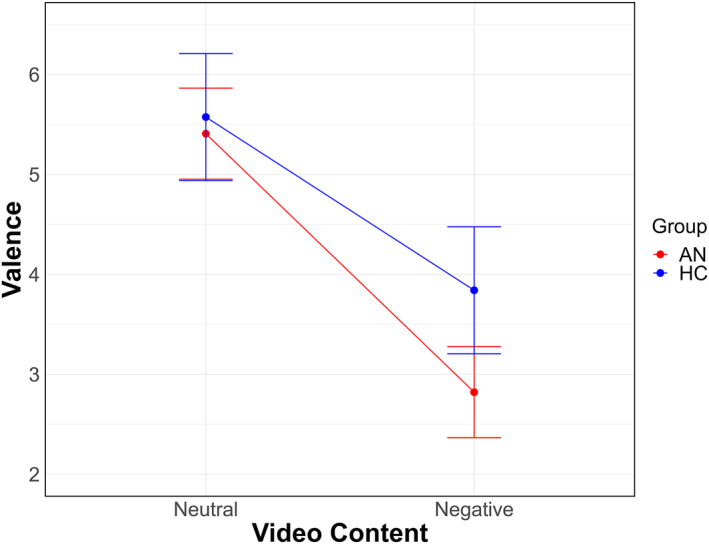
Differential effects of negative social evaluation on affective valence in patients with anorexia nervosa and healthy controls. AN is patients with anorexia nervosa; HC is healthy controls.

The LMM on valence showed significant main effects for the variables ‘Video Content’ [*X* ^2^ (1) = 83.62, *p* < 0.001] and ‘Group’ [*X* ^2^ (1) = 6.51, *p* = 0.012]. Overall, patients reported greater negative affect compared to controls and all participants experienced greater negative affect after viewing the negative videos compared to the neutral ones (Figure 2). The ‘Group by Video Content’ interaction was not significant [*X* ^2^ (1) = 3.13, *p* = 0.078].

#### 3.2.2 Wanting

**FIGURE 3:**
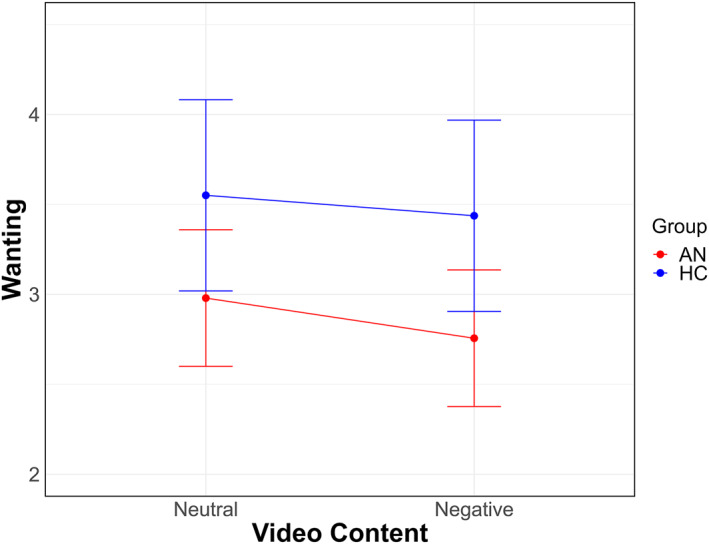
Differential effects of negative social evaluation on food wanting in patients with anorexia nervosa and healthy controls. AN is patients with anorexia nervosa; HC is healthy controls.

The LMM on wanting showed a significant main effect of ‘Group’ [*χ* ^2^ (1) = 4.17, *p* = 0.041], and “Video Content” [*χ* ^2^ (1) = 5.08, *p* = 0.024]. Overall, patients reported lower wanting for high calorie foods compared to healthy controls. Additionally, all participants reported lower wanting after viewing the negative videos compared to the neutral ones (Figure 3). The ‘Group by Video Content’ interaction was not significant [*χ* ^2^ (1) = 0.42, *p* = 0.517].

#### 3.2.3 Liking

The LMM on liking did not reveal any significant effects. The analysis showed no significant main effect of ‘Group’ [*χ* ^2^ (1) = 1.54, *p* = 0.215] or ‘Video Content’ [*χ* ^2^ (1) = 1.95, *p* = 0.162] nor a significant ‘Group by Video Content’ interaction [*χ* ^2^ (1) = 0.40, *p* = 0.533]. There were no significant differences in the liking of high‐calorie foods between patients with anorexia nervosa and healthy controls, nor between the negative and neutral video content conditions.

### 3.3 Relationship Between BMI, Eating Disorder Psychopathology, Social Anxiety Symptoms and Changes in Wanting and Liking of High‐Calorie Foods in Patients

Correlational analyses for the patient group are displayed in Table 2. The results indicated that patients with higher BMI presented a larger reduction in wanting (*ρ* = −0.430, *p* = 0.004) and liking (*ρ* = −0.391, *p* = 0.011) for high‐calorie foods following the negative videos. Patients with greater social anxiety reported a greater reduction in wanting following the negative videos (*ρ* = −0.364, *p* = 0.016). No other significant associations were found among the variables.

**TABLE 2:** Spearman's rho correlation.

Variable	Delta wanting	Delta liking	Delta valence
BMI (kg/m^2^)^a^	−0.430**	−0.391*	−0.220
EDE‐Q global score	0.047	0.174	−0.257
LSAS total score	0.210	0.248	−0.364*
Delta valence	−0.364	−0.032	—

*Note: N* = 43.

Abbreviations: BMI, body mass index; EDE‐Q, eating disorder examination questionnaire; LSAS, liebowitz social anxiety scale.

^a^ *N* = 42.

### 3.4 The Impact of Negative Social Evaluation on Wanting and Liking of High Calorie Foods in Moderately and Extremely Underweight Patients With Anorexia Nervosa

#### 3.4.1 Wanting

**FIGURE 4:**
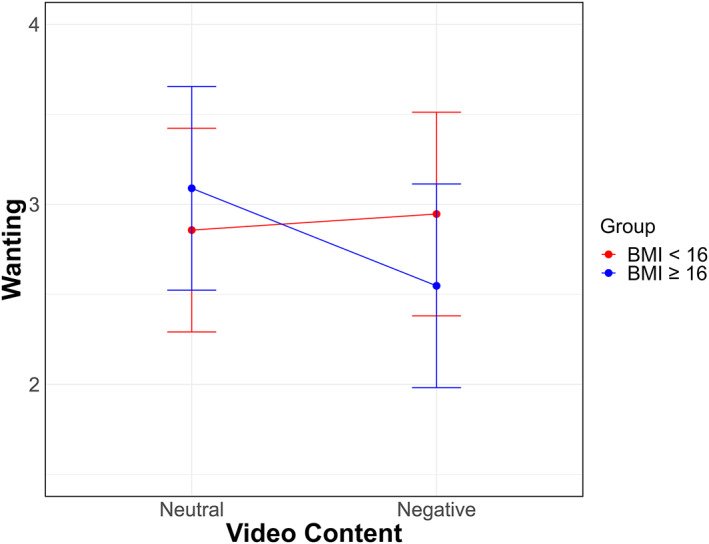
Differential effects of negative social evaluation on food wanting in severely and moderately underweight patients with anorexia nervosa. Patients with anorexia nervosa severely underweight (BMI < 16) and moderately underweight (BMI ≥ 16). BMI is Body Max Index (kg/m^2^).

The LMM on wanting revealed a significant ‘Video Content by Group’ interaction [*χ* ^2^ (1) = 9.65, *p* = 0.002], indicating that the effect of video content on food wanting varied by group (Figure 4). There were no significant main effects of ‘Video Content’ [*χ* ^2^ (1) = 0.39, *p* = 0.534] or ‘Group’ [*χ* ^2^ (1) = 1.01, *p* = 0.316]. Only moderately underweight patients showed a reduction in food wanting following the negative videos [*t* (40) = −3.77, *p* < 0.001]. No differences in food wanting based on type of video were observed among extremely underweight patients [*t* (40) = 0.62, *p* = 0.538]. Additionally, there were no significant differences between groups within each condition [Negative videos: *t* (45.6) = 1.00, *p* = 0.321; Neutral videos: *t* (45.6) = −0.58, *p* = 0.562].

#### 3.4.2 Liking

The LMM on liking did not reveal any significant effects. The analysis showed no significant main effect of ‘Group’ [*χ* ^2^ (1) = 0.13, *p* = 0.714] or ‘Video Content’ [*χ* ^2^ (1) = 0.01, *p* = 0.961] nor a significant ‘Group by Video Content’ interaction [*χ* ^2^ (1) = 2.94, *p* = 0.086]. Liking of high‐calorie foods did not differ significantly between patients who were extremely underweight and those who were moderately underweight, nor between the negative and neutral video content conditions.

## 4 Discussion

The aim of the present study was to assess whether inducing a negative emotional state, through exposure to videos of social rejection and criticism, would affect the wanting and liking for high‐calorie food pictures in patients with anorexia nervosa. The results on the affective valence scores confirmed the efficacy of the experimental manipulation. Following the observation of negative videos, both patients and controls displayed a significant decrease in their affective state, as compared to when neutral videos were displayed.

The effects of negative emotional states on food appraisal were also examined. In line with the hypotheses, the induction of a negative emotional state was associated with a significant reduction in food wanting in participants with anorexia nervosa. This is consistent with observational studies reporting an association between negative affect and increased calorie restriction in this patient group (Engel et al. 2013). This finding is particularly relevant from a clinical perspective, because it highlights the importance of social and environmental factors in influencing reward system responsiveness and implicitly affecting symptoms' severity and presentation. Not only patients, but also healthy controls exhibited a decrease in wanting for high‐calorie foods in situations of negative affect, which is in contrast with the initial hypothesis and with the broader literature on emotional eating (Cardi et al. 2015). However, it is important to note that not always individuals respond to negative situations with an increase in food consumption (Stijovic et al. 2025). Emotional eating is more common in individuals with disordered eating, body dissatisfaction, and psychopathological conditions (e.g. depression, anxiety) (Cardi et al. 2015; van Strien et al. 2016), all of which were exclusion criteria for our healthy control sample. Moreover, unlike previous protocols, in our study social exclusion was partially related to physical appearance, which may have elicited a qualitatively different emotional response—such as shame or body‐related distress—potentially leading to a reduced desire for food rather than the increase typically observed in general negative mood conditions.

Interestingly, the results revealed no significant modulation of ‘liking’ scores as a function of negative emotions. This lack of a significant difference in liking, despite clear effects on wanting, corroborates a dissociation between the two reward components, as already suggested by previous studies on emotional eating (Lemmens et al. 2011; Pool et al. 2015).

Finally, food wanting and liking in patients were correlated with clinical severity indices. Patients with higher BMI presented a larger reduction in both food wanting and liking compared to patients with lower BMIs. In particular, only moderately underweight patients showed a reduction in food wanting after viewing negative videos, whereas no such difference was observed among extremely underweight patients. A possible explanation is that patients with higher BMI might experience greater body dissatisfaction and insecurity and therefore they could be more sensitive to negative appearance‐related comments (Dang et al. 2023; Smith et al. 2017). However, we did not observe a significant association between BMI and the affective response to the negative videos, suggesting that the key difference between the two groups may not lie in the emotional impact per se, but rather in how this negative affect is managed. Specifically, patients with higher BMI might respond with an increased drive for dietary restriction—as indicated by the reduction in food wanting and liking—whereas patients with lower BMI may rely on alternative regulatory strategies. Although these hypotheses need further investigation, the observed finding highlights the importance of addressing interpersonal aspects during the treatment of anorexia nervosa. Negative social interactions in vulnerable individuals might reinforce illness‐related behaviours, negatively impacting therapy and leading to relapses and setbacks.

These findings need to be interpreted considering some limitations. The sample size was relatively small and the brief videos might have not represented the complexity of real‐life interactions. Larger and more ecological studies are needed to corroborate the observed pattern. Also, although age was included as a covariate in the analyses, patients with anorexia nervosa were significantly younger than healthy controls, which may still represent a residual confounding factor. Lastly, future studies should test the extent to which less wanting for high‐calorie foods might translate into increased restrictive behaviours, an hypothesis which this study did not test. Moreover, although the negative videos included both appearance‐ and personality‐related comments, our design was not powered to examine the differential effects of these two types of feedback. Future studies with larger samples and targeted designs could explore whether these dimensions elicit distinct responses across groups.

In conclusion, this study suggests that negative social interactions can exacerbate restrictive behaviours in patients with anorexia nervosa, particularly in those in recovery and at higher BMI. Psychological interventions to boost resilience to criticism and decrease interpersonal sensitivity might be particularly important during the weight recovery process.

## Conflicts of Interest

The authors declare no conflicts of interest.

## Data Availability

The data that support the findings of this study are available from the corresponding author upon reasonable request.

## Endnotes

^1^
IDs of selected pictures: 3, 20, 22, 25, 48, 68, 88, 106, 107, 145, 167, 310, 403.
